# Did My M.D. Really Go to University to Learn? Detrimental Effects of *Numerus Clausus* on Self-Efficacy, Mastery Goals and Learning

**DOI:** 10.1371/journal.pone.0084178

**Published:** 2013-12-23

**Authors:** Nicolas Sommet, Caroline Pulfrey, Fabrizio Butera

**Affiliations:** Social Sciences Institute, University of Lausanne, Lausanne, Switzerland; University of St Andrews, United Kingdom

## Abstract

Exams with *numerus clausus* are very common in Medicine, Business Administration and Law. They are intended to select a predefined number of academic candidates on the basis of their rank rather than their absolute performance. Various scholars and politicians believe that *numerus clausus* policies are a vector of academic excellence. We argue, however, that they could have ironic epistemic effects. In comparison with selective policies based on criterion-based evaluations, selection via *numerus clausus* creates negative interdependence of competence (i.e., the success of some students comes at the expense of the others). Thus, we expect it to impair students’ sense of self-efficacy and—by extension—the level of mastery goals they adopt, as well as their actual learning. Two field studies respectively reported that presence (*versus* absence) and awareness (*versus* ignorance) of *numerus clausus* policies at University was associated with a decreased endorsement of mastery goals; this effect was mediated by a reduction in self-efficacy beliefs. Moreover, an experimental study revealed that *numerus clausus* negatively predicted learning; this effect was, again, mediated by a reduction in self-efficacy beliefs. Practical implications for the selection procedures in higher education are discussed.

## Introduction

“Better to have an elite commando than an army of down-and-out”. This is how Guy Vallancien, urological surgeon and medical professor at the Université Paris Descartes, France, recently defended the use of *numerus clausus* in Medical Schools ([1] p 127). In many countries, students in Medical Sciences, Business Administration or Law often have to run competitive exams with *numerus clausus*, i.e., exams that select a fixed number or portion of candidates on the basis of their rank and not of their absolute performance [2,3]. As a function of the specific system implemented, *numerus clausus* determines which students are allowed to enter tertiary education, to proceed to the next academic year or to be given their diploma. Since their gradual implementation in the early 1960’s, *numerus clausus* policies have stirred up many controversies, such as the tensions between student unions and the Belgian government in Brussels [4], the 1976 political disputes concerning their constitutionality in Germany [5], or the 2005 European Court conviction of Austria for discriminative use of *numerus clausus* against non-native students [6]. 

Because of the wealth of militant, political and legal polemics, it is surprising that virtually no empirical studies—and literally no research in Social or Educational Psychology—have been devoted to the motivational and epistemic consequences of *numerus clausus*. Yet, longitudinal studies have reported that motivation toward learning in college is of paramount importance, as it predicts positive academic outcomes all through the curriculum [7] and positive career outcomes after graduation [8]. Thus, in this article, after defining *how* and *why* higher education institutions use *numerus clausus*, we will focus on the as yet unaddressed question of *what* the effects of such a policy are on students’ motivation and learning. 

### *How* does higher education make use of *numerus clausus*?

There exist two systems of selecting students via *numerus clausus*. On the one hand, candidates might be selected *upstream* from their entrance in higher education institutions. In this research, we designate this policy as *pre-curriculum numerus clausus*. For instance, this system is used in Florida, where admission in state universities is guaranteed for students reaching the top twenty percent of their high school graduation (i.e., “Talented Twenty Program” [9]).

On the other hand, candidates might be selected *downstream* from their entrance in higher education institutions. We designate this policy as *in-curriculum numerus clausus*. In such a case, *numerus clausus* can either be defined as *absolute* (i.e., a predefined number or percentage of acceptances to proceed to the next semester/year or obtain a diploma; in other words, the best X or the top X% of students pass the selection exam), or *flexible* (i.e., a predefined *range* of numbers or percentage of acceptance to proceed to the next semester/year or obtain a diploma; in other words, the best X ± Y or the top (X ± Y)% students pass the selection exam). The former scenario applies for example in the training of French primary and secondary schoolteachers [10]. At the end of their teaching training, students have to pass an absolute *numerus clausus* national recruitment exam so as to become certified teachers (e.g., in 2012, among the 1830 physical education teacher candidates, the government delivered 600 accreditations to the highest ranking ones [11]). 

In the latter scenario—flexible *numerus clausus*—the number of acceptances is fixed in advance but, depending on the year, a few students more or less can be allowed in. The use of flexible *numerus clausus* can be explicitly specified (e.g., see Johannes Gutenberg University of Mainz’s online FAQ, http://www.politik.uni-mainz.de/politikmaster/?page_id=122&lang=en) or implicitly stated (e.g., “waiting lists which act as a hidden *numerus clausus*” ([12] p 45). This last case appears to be widely used in higher education; for instance, De Paola [13] showed, on a sample composed of about 26,000 Italian students, that departments facing an excess of supply over demand (e.g., Physics, Chemistry, Mathematics) tended to reduce their academic standards and over-grade students (“grade deflation” or “easy grading practice”), whereas departments facing an excess of demand over supply (e.g., Law and Economics, Business Administration, Biology) tended to raise their academic standards and under-grade students (“grade inflation” or “hard grading practices”). In line with this phenomenon, Kaufman [14] reported that, in the United States, two-thirds of Law schools admitted to standardizing grades, and to doing it more strictly when students are more numerous. Although officious, grading practices at University therefore tend to be *relative* (depending on the ranking of a given student at the exam(s)), rather than *absolute* (depending on the performance of a given student at the exam(s)). In highly demanded fields, this mechanism contributes to regulate the stream of students allowed to pursue their curriculum, working as a hidden flexible *in-curriculum numerus clausus*. 

In summary, selection via *numerus clausus* could operate at two levels, namely *before* higher education entrance (i.e., *pre-curriculum numerus clausus*) or *after* higher education entrance (i.e., *in-curriculum numerus clausus*). As far as the second case is concerned, *in-curriculum numerus clausus* could either be *absolute* (pre-established number of admissions) or *flexible* (pre-established range of number of admissions). Due to the important prevalence of relative grading practices and grade standardization driven by demand and supply [15] our research will focus on flexible *in-curriculum numerus clausus* selection process.

### Why does higher education institution make use of *numerus clausus*?

Higher education institutions may defend the application of *numerus clausus* policies for two sets of reasons: practical and ideological. As far as practical reasons are concerned, *numerus clausus* policies firstly ensue from demographic trends. Due to population growth, the percentage of 19-21 years old entering European Universities indeed doubled from the 1960s to the 1970s [5] and again increased during the 1990s [16]. This generalized phenomenon, known as the “explosion of numbers” ([17] p 302) triggered the implementation of the first *numerus clausus* selection policies. Secondly, also concerning practical reasons, enrolment capacity appears as a major concern. For example, recent research has pointed out that in some universities the average number of students per faculty member can range from 24 to 400 in “over-enrolled disciplines” (e.g., University schools of Medicine ([3] p 146). Various higher education institutions therefore respond to this imbalance by applying highly selective admission procedures via the *numerus clausus*. Thirdly, governments may decide to regulate access to higher education in order not to train more workers than needed by society (i.e., adjusting labor supply to market demand [18]). Lastly, states might enforce drastic selective procedures for economic reasons (as an example, Switzerland spends, on average, 60’000 CHF (≈ 42’000 USD in 1997) per medical student during his/her first year of study [19]).

In addition to these practical reasons, policy-makers in higher education institutions may justify the use of *numerus clausus* with ideological arguments. Indeed, on the one hand, *numerus clausus* might be perceived as a primary vector in the search for excellence. For instance, some argue that French medical universities should maintain *numerus clausus* as it is because “medical doctors' added value lies on its scarcity” [20]. On the other hand, *numerus clausus*, could reflect a competitive culture based on meritocracy where students “who succeed and enter the second year [or proceed to the next semester or acquire their diploma] are considered to be heroes, the victors of a “war” that has defeated 9 out of 10 of their classmates” ([2] p 334). As inherited from a republic-based tradition of meritocracy of Western societies, *numerus clausus* policies could thus be perceived as consisting of a tough initiation rite and/or an academic vocation testing [21]. 

Practical and ideological reasons may conflict with each other. In French Medical Schools, for instance, where a state-controlled *in-curriculum numerus clausus* is established between the first and the second year, the number of allocated places diminished from mid-1980s to late-1990s mainly because of physician oversupply and expenditure reduction plans [22]. Such a reduction ultimately resulted in a shortage of healthcare professionals starting from 2008 [23]. Although demographers urge policy-makers to relax the limit of *numerus clausus* [24], they face a certain political reluctance. For example, Valerie Pécresse, the former French Minister for Higher Education and Research defended the decision to maintain *numerus clausus* by declaring that it “guarantees the quality of [France’s] health care system, the recruitment of the best and the more capable” [25]. As opposed to the practical / objective reasons (e.g., matching supply with demand), such ideological / subjective reasons (i.e., pursuing academic excellence) are not substantiated by any empirical research. Thus, in this article, we question the ideological posture that *numerus clausus* could be associated with some educational benefit, and aim at testing the effects of *numerus clausus* on students’ motivation and learning.

### *What* is the impact of *numerus clausus* on motivation and learning among higher education students?

#### *Numerus clausus* through the prism of Social Interdependence Theory

As opposed to social independence—where individuals may attain their goal regardless of others’ actions—social interdependence defines situations where: i) the members of a group share common goals; and ii) the accomplishment of each member’s goal is affected by others’ action [26]. More specifically a *negative* interdependence exists when the (perceived) probability of a group member reaching his/her goal decreases as other members also reach their goals (i.e., competition [27,28]). We argue that *numerus clausus* corresponds to negative interdependence at two levels: institutional and psychological.

Firstly, at the institutional level, *numerus clausus* implies a negative interdependence of rewards [29], be they admission to the next semester/year or delivery of certificates of graduation. Indeed, the number of resources being predefined (N places/diplomas are available), the fact that student A passes his/her examination automatically reduces the chances of student B to succeed (N – 1 places/diplomas are available). It is worth noting that—as *numerus clausus* is used most of the time in prestigious fields, disciplines or programs—negative interdependence of rewards additionally creates a negative interdependence in opportunities for upward social mobility (i.e., interdependent access to prestigious socio-professional positions). Furthermore, it should be noted that—due to the importance of the (negatively interdependent) rewards in terms of academic and professional achievement—*numerus clausus* clearly differs from constructive competition [30], as the first condition for competition to be constructive, namely the fact that “winning is relatively unimportant”, is not fulfilled ([31] p 323).

Secondly, at the psychological level, *numerus clausus* implies negative interdependence of the perceived competence of oneself and others [32]. In an *evaluative* context with no *numerus clausus* (i.e., where the selection criterion relies on a criterion-referenced standard, e.g., to reach an average of 80% of correct responses), students’ academic competence (AC) is defined in an absolute way: it solely corresponds to their own performance (OwP) at the final exam(s) (AC = OwP, i.e., social independence of competences). In a *selective* context with *numerus clausus* (i.e., where the selection criterion relies on a socially-referenced standard, e.g., to rank among the 20% best students), students’ academic competence (AC) is defined in a relative way: it depends on their own performance (OwP) at the final exam(s) but also on others’ performance (OtP) at the same exam(s) (AC = OwP / (OwP + OtP), i.e., social interdependence of competences). In such a case, the perception of one’s own competence necessarily comes into conflict with the perception of others’ competence, which, we argue, may yield deleterious motivational and/or epistemic effects. Why is it so?

#### *Numerus clausus*, self-efficacy and learning

Bandura defines perceived self-efficacy as a “judgment of how well one can execute courses of actions required to deal with prospective situations” ([33] p 122). In other words, self-efficacy corresponds to the belief that one is capable enough to master a specific task and to obtain positive results. As argued above, *numerus clausus* implies that academic success relies on both endogenous and relatively controllable factors (i.e., own performance), *and* exogenous and relatively non-controllable factors (i.e., others’ performance). The extent to which one perceives control over his/her behavior (i.e., *outcome* expectations: perceived likelihood that performing the behavior will produce a given outcome [34]) refers to *systemic* constraints (defined at macro-level), whereas the extent to which one perceives self-efficacy in his/her behavior (i.e., *efficacy* expectation: perceived ability to perform a behavior [34]) refers to *individual* beliefs (defined at micro-level [35]). In achievement contexts, low perceived control over exam performance due to external constraints has been found to deplete self-efficacy beliefs [36]. This reasoning echoes the literature on the link between social interdependence and psychological health, suggesting that negative interdependence increases individuals’ feeling of uncertainty [37] and reduces self-worth [31]. Thus, in this research, we argue that the very fact that the result of an exam with *numerus clausus* is determined by partially uncontrollable factors may reduce self-efficacy beliefs. In sum, our first hypothesis is that an evaluative system in which *numerus clausus* is implemented should induce lower self-efficacy beliefs than an evaluative system in which *numerus clausus* is not implemented.

According to expectancy-value theory [38], individuals regulate their motivation with the expectation that their behavior will lead to certain valuable outcomes. Because people act on what they think they are capable of, self-regulation of motivation and goal setting are notably regulated by self-efficacy [39]. In the realm of achievement, mastery goals—defined as the desire to acquire knowledge [40]—are the ideal theoretical construct to capture individual differences in motivational orientation toward learning. Scholars have consistently shown that high self-efficacy is associated with the pursuit of elevated mastery goals [41-43]. Recent studies using path modeling [44,45] showed that self-efficacy was an antecedent of the adoption of mastery goals—and not the reverse.

In this research, we argue that, when a task is perceived as having unknown scoring criteria—as is implied by *numerus clausus*—it will undermine the individual’s sense of efficacy, which in turn should impair mastery goal adoption. Thus, our second hypothesis is that, as opposed to an evaluative system with no *numerus clausus*, an evaluative system with *numerus clausus* will reduce mastery goal endorsement, and that this relation should be mediated by the reduction in self-efficacy experienced in the *numerus clausus* system when compared with the non *numerus-clausus* system.

In addition to the increase in the level of motivation toward learning (i.e., mastery goals), self-efficacy has been found to predict learning *per se* [46]. Key indices accounting for cognitive progress, such as choice of challenging task, elevated level of effort and task persistence, have indeed been shown to be sustained by high levels of self-efficacy (for a review, see 47). During task completion, self-efficacy is beneficial for both cognitive (more efficient self-monitoring) and conative (more adaptive learning strategy) outcomes [48]. For instance, using structural equation modeling, Sins and collaborators [49] showed that self-efficacy was associated with deep (vs. surface) cognitive processing and metacognitive strategies, which in turn were associated with achievement. Drawing the conclusions of two decades of research, Zimmerman stated that results “have clearly established the validity of self-efﬁcacy as a predictor of students’ […] learning” ([47] p 89). Thus, our third hypothesis is that, as opposed to an evaluative system without *numerus clausus*, an evaluative system with *numerus clausus* will reduce learning and that this reduction of learning should be mediated by the reduction in self-efficacy experienced in this context. 

## Hypotheses and Overview

Consequently, the aim of the present set of studies is to test the relationship between *numerus clausus* and both learning orientation and actual learning as mediated by self-efficacy. The first two studies take the form of field research, in which *numerus clausus* is respectively a natural group variable (i.e., academic sections not subject *versus* subject to *numerus clausus* policies) and a measured variable (i.e., medical students not perceiving *versus* perceiving their curriculum to be characterized by *numerus clausus* policies). Study 3 takes the form of experimental research in which *numerus clausus* is manipulated (i.e., absence *versus* presence of quota). In all three studies, we expect *numerus clausus* to lead to a reduction in self-efficacy beliefs (hypothesis 1). In Studies 1 and 2, we hypothesize *numerus clausus* to be negatively associated with mastery goals, a relationship mediated by self-efficacy (hypothesis 2). Study 3 goes further as it tests the deleterious effect of *numerus clausus* on learning, hypothesizing that this relationship should be mediated by self-efficacy (hypothesis 3).

## Study 1

### Method

#### Ethics Statement for the three studies

Neither medical, nor health related experimentation was performed. All Studies were conducted at University of Lausanne (Study 1 and 2) and/or at the Swiss Federal Institute of Technology in Lausanne (Study 1 and 3), Switzerland. 

Concerning Study 1 and 2—where no external intervention was implemented—experimenters followed the APA Ethical Guidelines for Research (http://www.sandplay.org/pdf/APA_Ethical_Guidelines_for_Research.pdf). Participants were informed that the study consisted in an anonymous survey on students’ academic profiles, and were entitled to decline or withdraw from participation. Following the completion of the questionnaire, participants were debriefed and were invited to ask any question about the research. 

Concerning Study 3—where the assessment mode of the alleged Internet test was manipulated—experimenters followed the same guidelines. In addition, all participants received a debriefing e-document (http://demosq4.free.fr/Quota/Information%20sur%20l '%C3%A9tude.pdf) in which particular attention was paid to ensure that participants could easily understand the nature and the aim of the manipulation. Moreover, they were invited to ask any additional questions about the research.

At the time of the three studies (2010-2012), no approval was needed in Switzerland to conduct research on human subjects. As stated by the Federal Administration of the Swiss Confederation (http://www.bag.admin.ch/themen/medizin/00701/00702/07558/index.html?lang=fr), the law relating to research on human subjects (i.e., constitutional article n°118b) will come into effect in January 1^st^ 2014. Given this legislation, the present research project was not submitted to any research ethics board.

#### Data availability for the three studies

The raw data for the three studies, as well as the IBM^®^ SPSS^®^ (version 19.0) syntax files used in conducting the analyses, are available from the authors upon request.

#### Participants

**Figure 1:**
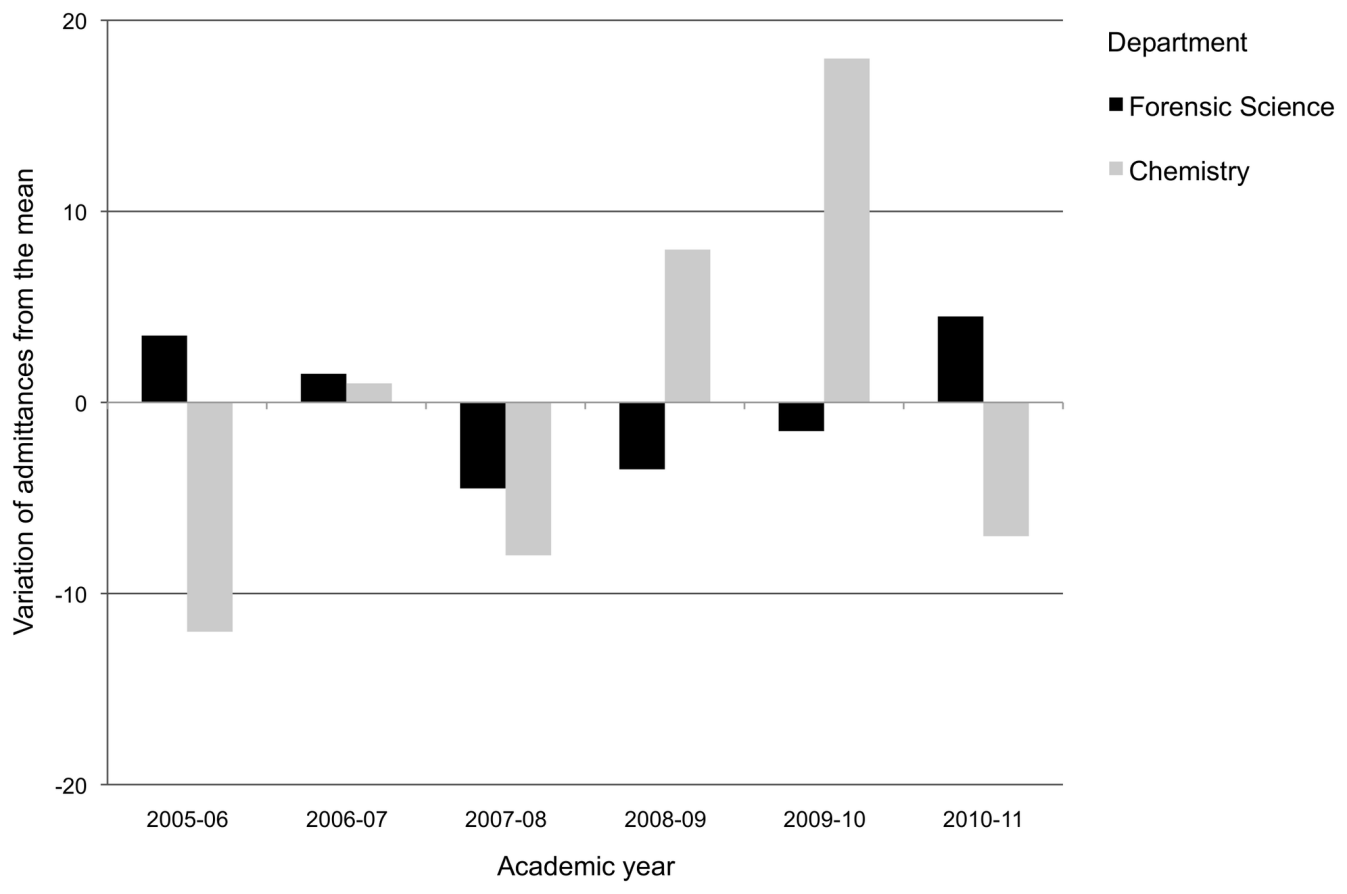
2005-2011 variations from the mean of the number of second year admittances for Forensic Science and Chemistry students (Study 1). Sources: University of Lausanne (http://www.unil.ch/statistiques) and Swiss Federal Institute of Technology in Lausanne (http://ogif.epfl.ch/).

Ninety first-year bachelor students from a French-speaking mid-size Swiss university participated in Study 1. Twenty non-Swiss respondents were dropped from the analyses (We decided to remove foreign students from Study 1 and 2’s samples as: i) Chirkov et al. [50] report that reasons to study abroad are plural (situation in home country, relationship with family, future career goals) and, thus, may be more relevant in an achievement goal setting than *numerus clausus* policies; ii) international students may not necessarily be fully aware of administrative and institutional aspects of the selection process). The final sample consisted of 40 women and 30 men with a mean age of 19.7 years (SD = 1.34). Forty-three of these undergraduates were enrolled in Chemistry and 27 in Forensic Science. These two sub-samples were chosen because students of the two Faculties have the same first-year curriculum, with the exception of two modules (i.e., Forensic Science and Criminology). Indeed, they share more than eighty percent of their courses, and therefore their overall academic profiles are comparable. Despite this similarity, the conditions of admission to second year are different. First-year Chemistry students must sit a final selective exam and obtain a pass grade of 4/6; in other words, these students are not subject to in-curriculum *numerus clausus* policies. On the contrary, first-year Forensic Science students must sit a final selective exam and place themselves among the top 15 ± 4 best candidates (based on department training capacity, number of acceptances is fixed in advanced, but—depending on the year—four more or four less students can be accepted); in other words, these students are subject to a flexible *in-curriculum numerus clausus* policy). As shown in Figure 1, variation in the number of admittances in second year from 2005-2006 to 2010-2011 is higher for Chemistry students, *SD* = 9.00 (for ≈ 81 candidates), than that of the Forensic Science students, *SD* = 3.17 (for ≈ 95 candidates), showing that the regulation of the flow of passing students for Forensic Sciences is a reality and not just a rule. Thus, proceeding to the second year depends on personal results for Chemistry students, whereas it depends on both personal and other students’ results for those in Forensic Science.

#### Procedure

The study was carried out during the Spring Semester, as students approached final exams. Chemistry and Forensic Sciences students attending the same biology class were invited to fill in a questionnaire, presented as a survey on “students’ academic profiles”. All students present at the course agreed to participate. Following the completion of the questionnaire, participants were debriefed.

### Measures

#### Self-efficacy

First, the questionnaire assessed students’ perceived self-efficacy using the French translation [51] of Midgley et al.’s self-efficacy scale ([52] p 20). Eight items required participants to indicate to what extent they felt efficient in their courses (e.g., “I can do almost all the work in class if I don't give up”). The measure ranged from 1, “not at all”, to 7, “*completely*” (α = .94, *M* = 4.57, *SD* = 1.18).

#### Mastery goals

Students’ endorsement of mastery goals was assessed using the French validation [53] of Elliot and McGregor’s Achievement Goal Questionnaire [54]. The three mastery goal items were extracted from the scale (e.g. “I want to learn as much as possible from the classes”). The measure could range from 1, “not at all”, to 7, “*completely*” (α = .84, *M* = 5.18, *SD* = 1.32).

#### Perception of *numerus clausus* policies

Students’ perception of the *numerus clausus* policies used in their department was assessed, as a check of the independent variable. Participants had to indicate to what extent, in their department, they thought “there is a limited number of places for the next year”. The measure could range from 1, “not at all”, to 7, “*completely*” (*M* = 3.64, *SD* = 2.41).

### Results

#### Check of perception of *numerus clausus* policies

In a preliminary analysis, we run a simple regression analysis on perception of *numerus clausus* policies with department as predictor (coded “-.5” for Chemistry and “+.5” for Forensic Science). Results showed a significant large effect, *B* = 3.84, *F*(1, 68) = 106.55, *p* < .001, η^2^_p_ = .61. As one should expect given the actual differences in policies, first-year Forensic Science students perceived to a greater extent that the number of places for the next year in their department was limited (*M* = 6.00, *SD* = .30) than did first-year Chemistry students (*M* = 2.16, *SD* = 0.23).

#### Overview of the linear regression analyses

**Figure 2:**
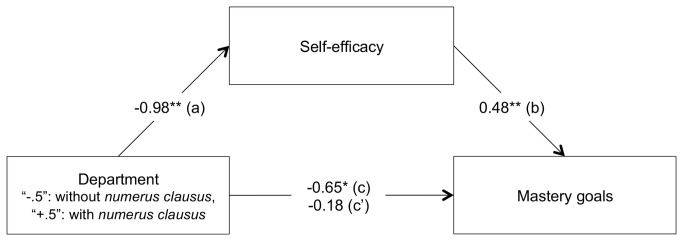
Mediation by self-efficacy of the relationship between academic department (with *versus* without *numerus clausus*) and mastery goals (Study 1). All values represent unstandardized coefficients. * *p* < .05, ** *p* < .01.

Linear regression analyses were conducted in two stages. In the first stage, we aimed at testing the direct effect of the department (coded “-.5” for Chemistry and “+.5” for Forensic Science) on mastery goals (c path). In the second stage, mediation analyses were carried out to test whether self-efficacy could explain this relationship (a, b and c’ paths). In preliminary analyses, a complete analysis of covariance [55] was conducted with gender (coded “-.5” for women and “+.5” for men) and centered age of the participants, as well as their interaction with the independent variable (i.e., department) on both mastery goals and self-efficacy. Because the inclusion of these terms was neither found to produce significant effects nor to alter the effects of the other variables, they were not included in further analyses. A summary of the results is presented graphically in Figure 2.

***Masterygoalorientation:*** A linear regression analysis was conducted with mastery goal adoption as the dependent variable and department as predictors. Results showed a significant effect of Department , *B* = -0.65, *F*(1, 68) = 4.16, *p* < .05, η^2^_p_ = .05 (c path). Forensic Science students (subject to *numerus clausus* policies) reported lower levels of mastery goal adoption (*M* = 4.78, *SE* = 0.25) than Chemistry students (not subject to *numerus clausus* policies, *M* = 5.43, *SE* = 0.20).

***Mediationalroleofself-efficacy:*** Then linear regression analyses were conducted to test the mediational role of self-efficacy in the relationship between department and mastery goal adoption. Firstly, a linear regression analysis with self-efficacy as dependent variable and department as predictors was conducted. Results showed that department negatively predicted self-efficacy, *B* = -0.98, *F*(1, 68) = 13.39, *p* < .001, η^2^_p_ = .16 (a path). In support of hypothesis 1, Forensic Science students (subject to *numerus clausus* policies) reported less self-efficacy (*M* = 3.97, *SE* = 0.21) than Chemistry students (not subject to *numerus clausus* policies) did (*M* = 4.94, *SE* = 0.17).

Secondly, linear regression analyses were conducted on mastery goals with department and self-efficacy as predictors. As predicted by hypothesis 2, results showed that the higher the level of self-efficacy experienced, the higher the degree of adherence to mastery goals, *B* = 0.48, *F*(1, 67) = 12.88, *p* < .001, η^2^_p_ = .16 (b path), while the effect of department on mastery goals became non-significant, *B* = -0.18, *F* < 1, *ns*, η^2^_p_ = .00 (c’ path). Preacher and Hayes’ bootstrap method [56] confirmed the significance of the indirect effect of Department on mastery goals through self-efficacy (ab path), *B* = -0.46, *SE* = .21, with a BCa 95% CI of -1.02 to -0.15, *R* ^2^_MED_ = .03. (*R* ^2^_MED_ designated the effect size of the indirect effect in term of explained variance. It was calculated using MacKinnon’s equation, *R* ^2^_MED_ = (*r* ^2^_*MX*_) (*r* ^2^_YM,X_) [57], where *r* ^2^_*YM*_ is the squared correlation of self-efficacy (M) with department (X), and *r* ^2^_YM,X_ is the squared partial correlation of mastery goals (Y) with self-efficacy (M), partialing out department (X) (see also, 58). Gender of the participants was systematically controlled. A similar procedure was used to calculate the effect size of Studies 2 and 3’s indirect effects.)

### Discussion

In line with our hypotheses, the results indicate that first-year students of a department subject to *numerus clausus* policies (i.e., with a final selective examination based on a socially-referenced standard) endorse mastery goals to a lesser extent than the students enrolled in a department where they are not subject to *numerus clausus* (i.e., with final selective examination). Moreover, as expected, this effect is mediated by perceived self-efficacy. Students enrolled in a department with *numerus clausus* report lower self-efficacy than those enrolled in a department with no *numerus clausus*. This depletion of self-efficacy therefore leads individuals to be less motivated to learn.

Although Study 1’s analysis on perception of *numerus clausus* policies clearly indicates that first-year Forensic Science students report being subject to *numerus clausus* to a larger extent than first-year Chemistry students, one might wonder whether it is *numerus clausus per se* that impacted their perceived self-efficacy and mastery goals. Indeed, one cannot exclude that the results are due to some confounding variable related to another difference between the two Faculties (e.g., specific academic socialization). In Study 2, we address this issue by testing the effect of the perception of *numerus clausus* in the same Department: the Medical School. 

## Study 2

We chose to conduct Study 2 with Medical School students because of an interesting characteristic. Despite the fact that the Dean’s Office formally denies the use of an official *numerus clausus* in their Department, first-year Medical School students at this University are known to have divergent opinions on the matter. Whilst some of them define the Medical School’s selective policies as being utterly *numerus clausus* (and there are actually rumors about these policies being formal), others do not. We took advantage of the ambiguity in this situation, and hypothesized that first-year Medical students who perceive that they are subject to *numerus clausus* in the selection for entry to their second year of studies should endorse lower levels of mastery goals than the ones who do not perceive this to be the case. As in Study 1, we expected this link to be mediated by self-efficacy.

### Method

#### Participants

One hundred and fifty-nine first-year Medical School students from a French-speaking mid-size Swiss university participated in Study 2. As in Study 1, nineteen non-Swiss respondents were dropped from the analyses. One participant, whose mastery goal score was more than 4 *SD* below the mean, was identified as an outlier with Tukey's procedure [59] and hence was also removed from the analyses. The final sample was composed of 96 women and 43 men with a mean age of 19.9 years (SD = 2.45).

#### Procedure

The study was carried out during the spring semester. At the beginning of an anatomy class, first-year Medical School students were given the same questionnaire as in Study 1. All students present at the course agreed to participate. Before filling the questionnaire in, participants were asked to indicate whether they reckoned or not that, “officially, there is a *numerus clausus* policy (quota) in [their] department”. Experimenters knew anecdotally the peculiarity that some of them considered their department’s selective policy as being *numerus clausus* while some others did not. This expectation was checked with the vice-Dean of education of the “Faculté de Biologie et Médecine”, who told that he often receives first-year Medical School students in his office complaining about the use of *numerus clausus*, while officially there is none. We were able to empirically confirm this anecdotal evidence, since 63% of the students of our sample stated that such policy applied to their department (*N* = 88) while 37% of them stated that it did not (*N* = 51). We used this differential perception of in-curriculum *numerus clausus* as our independent variable. Following the completion of the questionnaire, participants were debriefed.

### Measures

#### Self-efficacy and mastery goals

Students’ levels of self-efficacy (α = .89, *M* = 4.89, *SD* = .95) and mastery goal orientation (α = .68, *M* = 6.18, *SD* = 0.80) were measured using the same questionnaires as in Study 1.

### Results

#### Overview of the Regression Analyses

**Figure 3:**
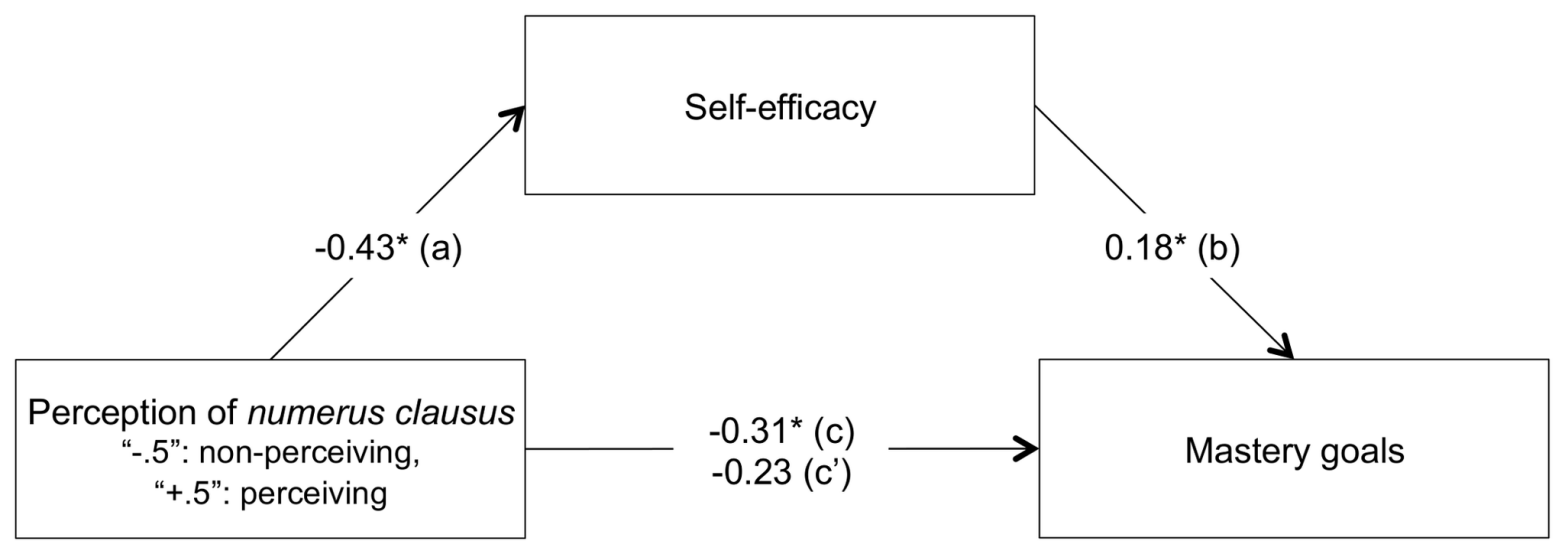
Mediation by self-efficacy of the relationship between perceived *numerus clausus* policies and mastery goals (Study 2). All values represent unstandardized coefficients. * *p* < .05, ** *p* < .01.

Linear regression analyses were once again conducted in two stages. In the first stage, we aimed at testing the direct effect of perception of in-curriculum *numerus clausus* (coded “-.5” for perception of no *numerus clausus* and “+.5” for perception of *numerus clausus*) on mastery goals (c path). In the second stage, mediation analyses were carried out to see if self-efficacy could explain this relationship (a, b and c’ paths). As in Study 1, in preliminary analyses, a complete analysis of covariance was conducted with gender (coded “-.5” for women and “+.5” for men) and centered age of the participants, as well as their interaction with the independent variable (i.e., perception of *numerus clausus*) on both mastery goals and self-efficacy. Because the inclusion of age, was neither found to produce significant effects nor to alter the effects of the other variables, it was dropped from the final analyses. Gender was found to yield a main effect on self-efficacy, and was therefore included as a covariate in further analyses. A summary of the results is presented graphically in Figure 3.

***Masterygoalorientation:*** In preliminary analyses, a Levene’s test revealed that variance of mastery goals significantly differed in function of the perception of *numerus clausus*, *F* (1, 135) = 8.23, *p* < .005. In order to relax the homoscedasticity assumption in linear regression, we used heteroscedasticity-robust estimator using the HC4 method [60] (see also 61). A heteroscedasticity-robust linear regression was therefore conducted on mastery goals with perception of *numerus clausus* and gender as predictors. Results showed a significant effect of perception of *numerus clausus*, *B* = -0.31, *F*(1, 136) = 3.93, *p* < .05, η^2^_p_ = .02 (c path): Students who perceived a *numerus clausus* policy in their department reported lower levels of adherence to mastery goals (*M* = 6.00, *SE*(HC) = 0.15) than those who did not (*M* = 6.31, *SE*(HC) = 0.08). No other effect reached significance.

***Mediationalroleofself-efficacy:*** Then, linear regression analyses were conducted to test the mediational role of self-efficacy in the relationship between the perception of *numerus clausus* and mastery goal adoption. Firstly, a linear regression analysis with self-efficacy as dependent variable and perception of *numerus clausus* and gender as predictors was conducted. Results showed that the perception of *numerus clausus* was negatively associated with self-efficacy, *B* = -0.43, *F*(1, 136) = 7.44, *p* < .008, η^2^_p_ = .05 (a path). In support of hypothesis 1, students who perceived there to be a *numerus clausus* policy in their department reported less self-efficacy (*M* = 4.74, *SE* = 0.13) than those who did not (*M* = 5.17, *SE* = 0.10). Unexpectedly, analyses also revealed a positive effect of gender, *B* = 0.60, *F*(1, 136) = 13.21, *p* < .001, η^2^_p_ = .08. Male participants were found to report more self-efficacy (*M* = 5.25, *SE* = 0.14) than female participants (*M* = 4.65, *SE* = 0.09).

Secondly, heteroscedasticity-robust linear regression analyses were conducted on mastery goals with perception of *numerus clausus* and self-efficacy as predictors. Results showed that the higher the degree of self-efficacy experienced, the higher the degree of adoption of mastery goals, *B* = 0.18, *F*(1, 135) = 6.19, *p* < .02, η^2^_p_ = .04 (b path), while the effect of perception of *numerus clausus* on mastery goals became non-significant, *B* = -0.23, *F*(1, 135) = 2.35, *p* = .13, η^2^_p_ = .01 (c’ path), as predicted by hypothesis 2. Hayes’ bootstrap method [62] aiming at calculating the indirect effect with heteroscedasticity-consistent standard error estimator confirmed the significance of the indirect effect of perceived *numerus clausus* on mastery goals through self-efficacy (ab path), *B* = -0.08, *SE*(HC) = 0.04, with a BCa 95% CI of -0.18 to -0.01, *R* ^2^_MED_ = .002.

### Discussion

Congruent with study 1, the present results reveal that first-year medical school students perceiving that they are subject to *numerus clausus* in their department endorsed less mastery goals than the ones who do not. Furthermore, as expected, this effect is mediated by perceived self-efficacy. These results provided convergent validity to the results of Study 1, as the present findings are obtained by focusing on the impact of *numerus clausus* in its psychological dimension among students of the same department. 

The unpredicted finding that men reported being more self-efficacious than women is of interest in that such a gender difference has often been reported in the literature (e.g., for medical students’ self-efficacy beliefs related to anatomy [63]). This phenomenon could also be explained by the fact that men tend to be more self-congratulatory when responding to this scale, while girls tend to be more modest [64].

In Studies 1 and 2, the *numerus clausus* variable respectively consisted of natural groups and measured perception. Although high in ecological validity, neither of these studies enables us to make causal inferences about the role of *numerus clausus*. In Study 3, the theoretical underpinnings of *numerus clausus* are therefore manipulated, namely the aforementioned distinction between a selection criterion based on an absolute standard (e.g., to obtain a given grade) and a selection criterion based on a normative standard (e.g., to obtain a given rank). Also, the desire to learn, measured by mastery goals, although considered by many as a precursor of learning, has been shown to yield an inconsistent relationship with actual learning (for an integrative review, see 65). Study 3 thus goes further by directly testing the effects of *numerus clausus* on learning, again with the hypothesis that this relationship should be mediated by self-efficacy.

## Study 3

### Method

#### Participants and design

Two hundred and thirteen students attending a Swiss polytechnical university that trains engineers and architects, volunteered to participate in Study 3. This sample was selected because this University does not have a *numerus clausus* policy, which could have interfered with the manipulation. One participant, whose learning test score was more than 4 *SD* below the mean, was identified as an outlier with Tukey's procedure [59] and was removed from the analyses. The final sample was composed of 66 women and 146 men with a mean age of 21.3 years (SD = 2.41), randomly assigned to two conditions, namely the *numerus clausus* condition (28 women and 78 men) and the non-*numerus clausus* condition (38 women and 68 men). All of them were students, namely 64 first-year, 52 second-year, 42 third-year bachelor’s students and 54 master’s students. It should be noted that in the university where the study was conducted—as in other universities within the countries that have undergone the so-called “Bologna process”— students have to complete a five-year curriculum to become engineers or architects, which makes the Master’s program the logical continuation of the Bachelor program. 

#### Procedure

**Figure 4:**
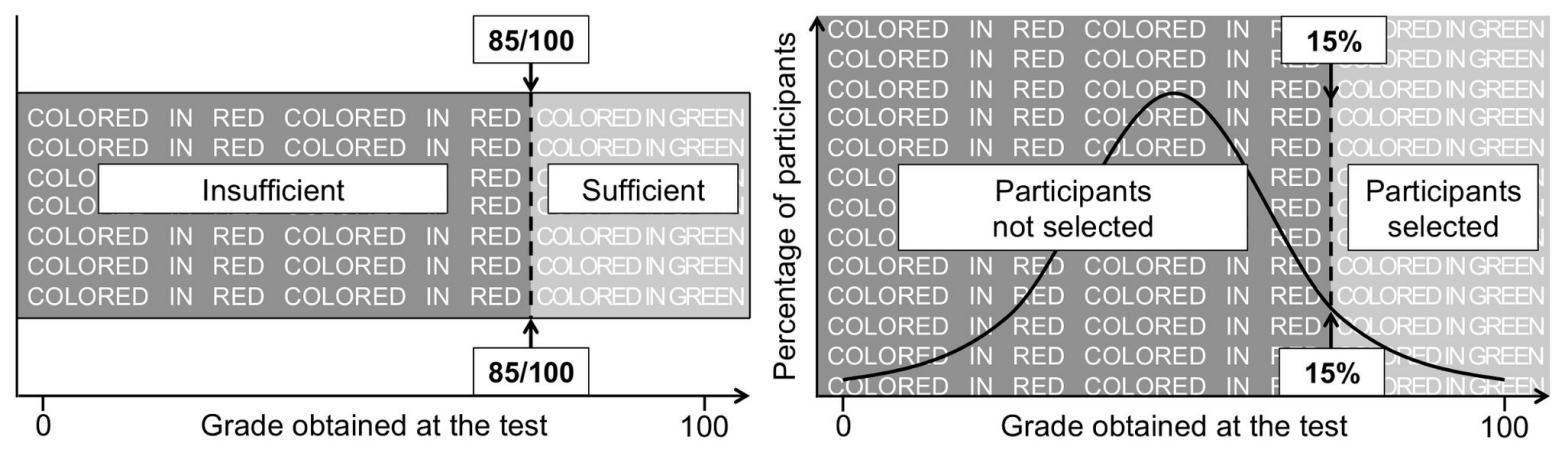
Presentation of the test as not having *numerus clausus* (left panel; criterion-based evaluation) or as having it (right panel; norm-based selection; Study 3).

**Figure 5:**
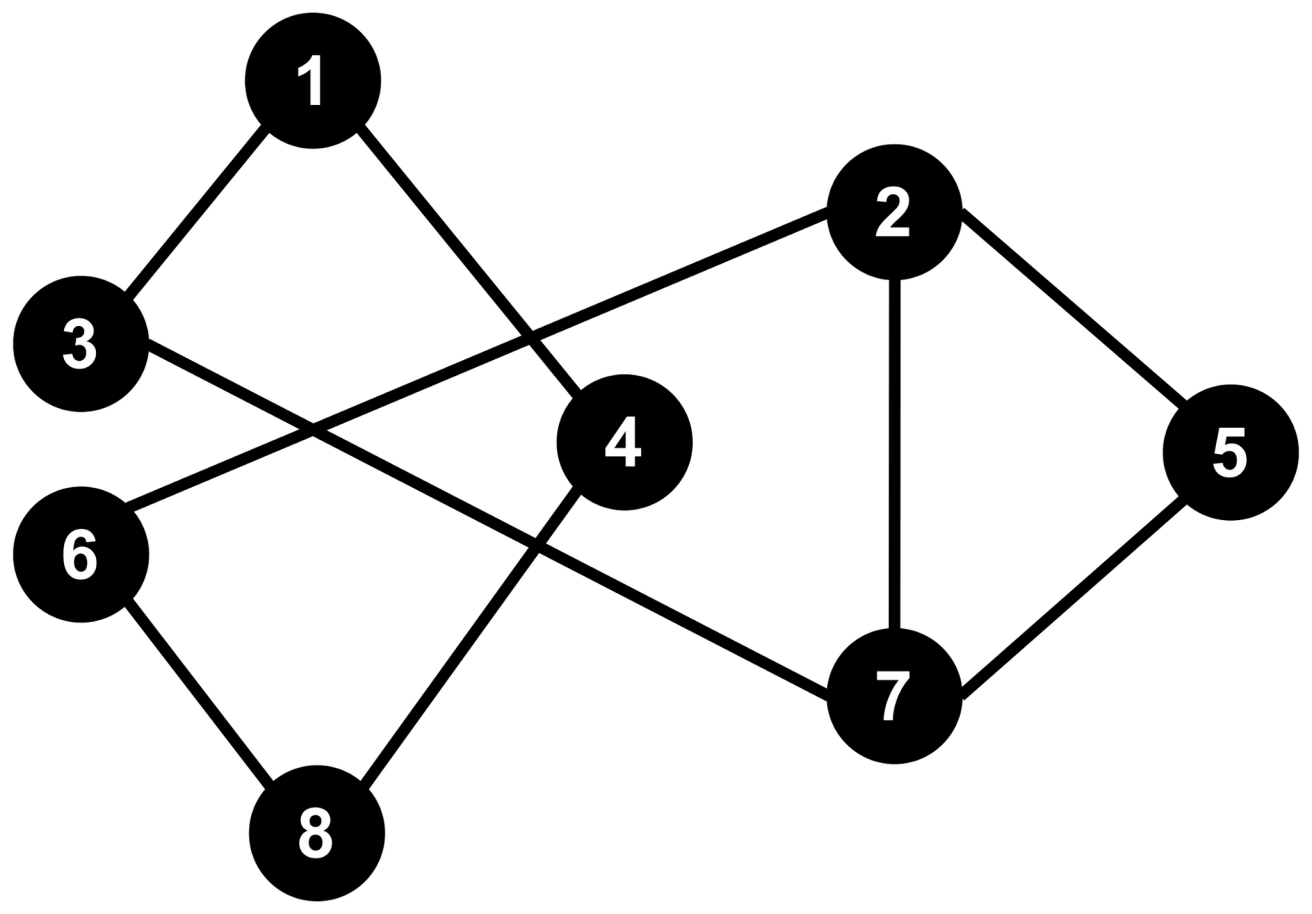
Example of one of the eight graph problems (Study 3).

The study was conducted on the Internet in the Spring semester. The experiment was introduced as the validation of a fictitious test, namely the “APT reasoning test”, designed to be diagnostic of individuals’ reasoning competences. As shown in Figure 4, we manipulated *numerus clausus* by presenting the test either as a criterion-based assessment tool aiming at evaluating individual’s competence in reasoning (i.e., in reaching competence standard, respondents have to obtain a score of 85/100 at the test, *N* = 106; “the non-*numerus clausus* condition”), or as a norm-based assessment tool aiming at selecting individuals competent in reasoning (i.e., in reaching competence standard, respondents have to be among the 15/100 best participants, *N* = 106; “the *numerus clausus* condition”). It is important to note that, in the *numerus clausus* condition, participants were not only explained that their performance would be positioned on this normally-distributed competence curve (as it is the case for norm-referenced tests [66]), but also that their position on the curve would determine whether or not they will be selected (as it is specifically the case of *numerus clausus* policies, but not of any norm-based assessment). Following the instructions, participants were invited to read a 500-word text introducing the graph problems of the test (for an example of a graph problem, see Figure 5). 

The text was concerned with graph theory. Among non-relevant information concerning the historical background and mathematical anecdotes, the text presented the principles for solving the test problems. However, these principles were given indirectly and participants had to identify and learn how to use them, so as to apply them to graph problems. In particular, the text presented as an adaptation of the classic Seven Bridges of Königsberg problem. It explained that the Pregel River divided Königsberg (now Kaliningrad) in four parts, connected by seven bridges. The Königsberg problem consists in finding out whether walking through the seven bridges only once and *coming back to one*’s *original position* is feasible. Formally, in graph theory, if this circuit exists, the graph is called Eulerian [67]. It was named after the mathematician Leonhard Euler, who showed that such a route existed if, and only if, *each part of the city had an odd number of bridges*. 

In order to perform well on the test, participants had to understand and to assimilate this principle so as to transfer it to the graph system: bridges of the Königsberg problem corresponded to the vertex of a graph, while parts of the city corresponded to its dots. A participant fully understanding Euler’s demonstration could therefore deduce that drawing a graph in one continuous stroke and finishing to the starting point is feasible if, and only if, *each dot of the graph has a odd number of lines* (principle related to question iii, see below). We rely here on a cognitive conception of learning, defined as an active process of acquiring knowledge and knowledge structure, so as to improve performance on a subsequent task [68]. Our task is therefore a learning task to the extent that most of the problems of the test were too complicated (i.e., graphs having numerous lines and dots) to be solved with a naïve trial-and-error approach, and without having understood the underlying principles.

The test itself contained eight graph problems. For each of these graphs, participants had to determine whether or not it was possible: i) to color dots by using only two colors knowing that two connected dots cannot share the same color (graph coloring); ii) to draw the graph in one continuous stroke without going over any line twice (i.e., a semi-Eulerian path [67]); iii) to draw the graph in one continuous stroke and finishing to the starting point without going over any line twice (i.e., an Eulerian graph). Four weeks later, participants were given their score at the test and fully debriefed.

### Measures

#### Self-efficacy

Students’ perceived self-efficacy during test completion (α = .94, *M* = 4.30, *SD* = 1.29) was measured using the same questionnaire as that used in Studies 1 and 2, adapted at task level (e.g., “While completing the APT Reasoning test […] I was able to solve almost all the [graph] problems if I made an effort”). It should be noted that assessment of self-efficacy beliefs was conducted after the task (i.e., before participants were debriefed); indeed, in experimental settings in which filling in a mediator scale might contaminate responses to the dependent variable by rendering participants suspicious, as in the present case, it is recommended to measure the mediator at the end [69].

#### Learning Score

Participants who truly assimilated the principles presented through the examples of the text should be able to apply this knowledge to the graph problems; thus, a short-term learning score was computed by summing the correct answers to the 3 questions x 8 graphs of the test. The score ranged from 14 to 24, with a mean of 21.64 (SD = 1.96).

### Results

#### Overview of the Regression Analyses

**Figure 6:**
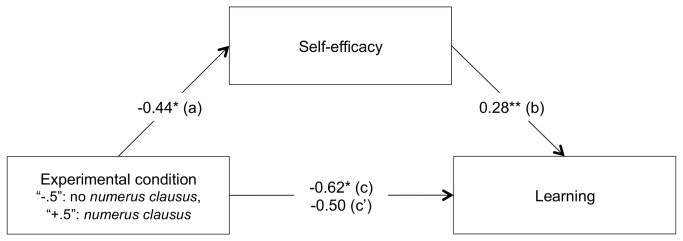
Mediation by self-efficacy of the relationship between the *numerus* *clausus* conditions (absence *versus* presence) and learning (Study 3). All values represent unstandardized coefficients. * *p* < .05, ** *p* < .01.

Linear regression analyses were conducted in two stages. In the first stage, we aimed at testing the direct effect of the experimental variable (coded “-.5” in the absence of *numerus clausus* and “+.5” in the presence of *numerus clausus*) on the learning score (c path). In the second stage, mediation analyses were carried out to see if self-efficacy could explain this relationship (a, b and c’ paths). As in Studies 1 and 2, in preliminary analyses, a complete analysis of covariance with gender (coded “-.5” for women and “+.5” for men), academic level (coded “-2” for first-year, “-1” for second-year, “1” for third-year bachelor’s students and “2” for master’s students), and centered age of the participants, as well as their interactions with the independent variable (i.e., manipulation of *numerus clausus*) was conducted on both learning score and self-efficacy. Because the inclusion of academic level and its interaction with the experimental independent variable was neither found to produce significant effects nor to alter the effects of the other variables, these terms were not included in further analyses. However, a main effect of gender (on the learning score and on self-efficacy), and two interaction effects between gender and *numerus clausus* and between age and *numerus clausus* (both on the learning score) were found. Thus, these terms were entered as covariates. The final model contained five predictors: the experimental variable, gender, age, gender x *numerus clausus* and age x *numerus clausus*. A summary of the most important results is presented graphically in Figure 6.

***Learningscore:*** Linear regression analyses were conducted on the learning score with the above model. Results showed a significant negative effect of the experimental variable, *B* = -0.62, *F*(1, 206) = 4.75, *p* < .04, η^2^_p_ = .02 (c path): In the presence of *numerus clausus*, participants obtained a lower learning score (*M* = 21.17, *SE* = 0.21) than in the absence of *numerus clausus* (*M* = 21.79, *SE* = 0.19). Unexpectedly, results also showed a significant positive main effect of gender, *B* = 0.71, *F*(1, 206) = 6.14, *p* < .02, η^2^_p_ = .02. Male students obtained a higher learning score (*M* = 21.84, *SE* = 0.16) than female students (*M* = 21.12, *SE* = 0.24). Additionally, the interaction between gender and the experimental variable was found to be significant, *B* = 1.31, *F*(1, 206) = 5.19, *p* < .03, η^2^_p_ = .02: The *numerus clausus* condition led to a significantly lower learning score than the non-*numerus clausus* condition among female students, *B* = -1.25, *F*(1, 206) = 6.81, *p* < .01, η^2^_p_ = .03, but not among male students, *B* = 0.05, *F* < 1, n.s. It is worth noting that the interaction between age and the experimental condition, although significant in the preliminary analysis, failed to reach significance in the final analyses, *B* = -0.20, *F*(1, 206) = 3.24, *p* = .08. No other result reached significance.

***Mediationalroleofself-efficacy:*** Linear regression analyses were conducted to test the mediational role of self-efficacy in the effect of the experimental variable on the learning score. Firstly, multiple regression analyses on self-efficacy were conducted with the experimental variable as predictor. Gender and age, as well as their interaction with the experimental condition were again entered in the model. Results showed that the experimental variable negatively predicted self-efficacy, *B* = -0.44, *F*(1, 206) = 5.83, *p* < .02, η^2^_p_ = .02 (a path). In support of hypothesis 1, in the presence of *numerus clausus*, participants reported less self-efficacy (*M* = 3.92, *SE* = .14) than in the absence of *numerus clausus* (*M* = 4.37, *SE* = .12). As in Study 2, results also showed a significant positive effect of gender, *B* = 0.81, *F*(1, 207) = 19.23, *p* < .001, η^2^_p_ = .08. Male students reported more self-efficacy (*M* = 4.55, *SE* = 0.10) than female students (*M* = 3.74, *SE* = 0.15).

Secondly, multiple regression analyses were conducted on the learning score with the experimental variable and self-efficacy as predictors. Gender and age, as well as their interaction with the experimental variable were once again entered in the model. As expected in hypothesis 3, results showed that the higher the self-efficacy, the higher the learning score, *B* = .28, *F*(1, 205) = 6.95, *p* < .01, η^2^_p_ = .03 (b path), while the effect of the experimental condition on learning became marginal, *B* = -0.50, *F*(1, 205) = 3.04, *p* = .09, η^2^_p_ = .01 (c’ path). The effect of gender also became non-significant, *B* = .48, *F*(1, 205) = 2.67, *p* = .11 η^2^_p_ = .01, whereas the interaction between gender and experimental variable remained significant, *B* = 1.29, *F*(1, 205) = 5.23, *p* < .03, η^2^_p_ = .02. Preacher and Hayes’ bootstrap method [56], *B* = -0.13, *SE* = 0.08, with a BCa 95% CI of -0.33 to -0.02, *R* ^2^_MED_ = .001, confirmed the significance of the indirect effect of experimental condition on the learning score through self-efficacy (ab path), giving support to hypothesis 3. It is worth noting that the indirect effect of gender on learning score through self-efficacy was also significant, *B* = 0.23, *SE* = 0.09, with a BCa 95% CI of .09 to .48, *R* ^2^_MED_ = .001.

### Discussion

By manipulating *numerus clausus* through its theoretical underpinnings—namely by presenting the test as either a criterion-based assessment tool aiming at evaluating individual’s competence or a norm-based assessment tool aiming at selecting competent individuals (therefore akin to *numerus clausus*)—the present experiment reveals that presenting an evaluative task as having *numerus clausus*, in comparison as having no *numerus clausus*, led to a decline in short-term learning, thereby extending the results of studies 1 and 2. It is worth noting that the total effect, although significant, was small in terms of effect size (i.e., .01 < η^2^_p_ < .06, [70]), which requires some caution in interpreting the relationship between *numerus clausus* and learning. However, consistently with studies 1 and 2, the detrimental effect of *numerus clausus* on learning was mediated by the depletion of the same mediating variable, namely self-efficacy beliefs. 

Additionally, as in Study 2, men in our sample reported more self-efficacy than women. However, this difference, although not part of our a priori hypotheses, may be explained with the existing literature. Our task emphasizes logical-mathematical intelligence, which is traditionally judged as being masculine [71]. Moreover, stereotypes drive gender differences in self-efficacy, with women reporting lower level of self-efficacy in mathematics [72] or computing abilities [73]. Finally, our results showed that this reduction in self-efficacy beliefs mediated the effect of gender on learning: Due to the stereotypically masculine nature of the task, women reported being less efficacious and—as a consequence—learnt less. These findings replicate those of Pajares and Miller [74] showing the mediational role of self-efficacy in the relationship between gender and mathematical problem solving.

Another interesting consideration related to gender is that the deleterious effect of *numerus clausus* on learning appears to be particularly pronounced for women. Although unpredicted, this finding is coherent with the literature on stereotype threat (for a review, see 75). As mentioned above, given the logical-mathematical nature of our task, women of our sample, in comparison to men, may have suffered from stereotype threat. In particular, the *numerus clausus* condition was operationalized by presenting the test as a norm-based assessment, which implied that they were in direct competition with other students of their university, that is to say a vast majority of *male competitors* (N.B., in 2011, 73% of students in the polytechnical university used in the study were men, http://ogif.epfl.ch/), in a field where they have a lower status than men (i.e., STEM, Science, Technology, Engineering, and Mathematics, [76]). Thus, the *numerus clausus* condition could have made the gender stereotype about mathematical ability even more salient and disrupted women’s learning [77].

## General Discussion

Since the 1960s, *numerus clausus* has been a source of recurrent ideological disputes, from student activism to parliamentary debates. In this regard, political or intellectual elites in favor of these selective policies often argued that they convey a culture of academic and professional excellence [1]. It is then quite surprising that only the impact of *pre-curriculum numerus clausus* (i.e., selection operating *before* entrance into higher education, e.g., [78,79]) has been empirically studied, and never that of *in-curriculum numerus clausus* (i.e., selection operating *after* entrance into higher education). Thus, the first contribution of the present research is to document, for the first time in Educational or Social Psychology, the detrimental effects of *in-curriculum numerus clausus* on motivation to learn and learning. 

This phenomenon was captured in three contexts: institutional, intra-individual and experimental. In Study 1, *numerus clausus* was a situational *institutional* variable (i.e., Department not applying vs. applying *numerus clausus*). Results showed that *numerus clausus* policies predicted a lower level of mastery goal endorsement through weakened self-efficacy beliefs. Then, the negative effect of *numerus clausus* on mastery goals was replicated with an *intra-individual* variable. Indeed, in Study 2, we captured *numerus clausus* through the subjective perception of students (i.e., Medical students not perceiving vs. perceiving the implementation of a *numerus clausus*). Results showed that *numerus clausus* perception also reduced the level of mastery goal endorsement through weakened self-efficacy beliefs. Finally, the deleterious effect of *numerus clausus* on learning was assessed in an *experimental* context. *Numerus clausus* was a manipulated variable: An evaluative test was presented as either criterion-referenced assessment (absence of *numerus clausus*, as everybody can succeed, provided sufficient performance), or norm-referenced assessment (presence of *numerus clausus*, as success requires to be in the top 15% of participants). Results showed that *numerus clausus* induction reduced actual short-term learning through weakened self-efficacy beliefs. In sum, both studies 1 and 2 have high ecological validity, and study 3 complements this by assessing the causal role of *numerus clausus*. 

The second contribution of the present research is that the mediational effect of self-efficacy allows us to understand why it is the very structure elicited by *in-curriculum numerus clausus* that reduces motivation to learn and learning. Indeed, the context of negative interdependence typical of *numerus clausus* provides a *post hoc* (vs. *pre hoc*) socially-referenced (vs. auto-referenced) standard, such that academic achievement is not only internally based (i.e., dependent on relatively controllable personal effort) but also externally based (i.e., dependent on the uncontrollable results of other students). Peers’ achievement being uncontrollable, *numerus clausus* may therefore impair the students’ beliefs about their ability to succeed. Accordingly, our results show that as self-efficacy decreases, the level of endorsement of mastery goals (Study 1 and 2) and actual learning (Study 3) decline.

In this respect, our results have important implications regarding the three-decade debate between the pro- and the anti-*numerus clausus* advocates. On the one hand, the ideological argument stating that *numerus clausus* elicits higher education excellence [80] is not supported by the data. Indeed, in Studies 1 and 2, we showed that, instead of being pushed to being interested in their discipline and in learning, students subjected to *numerus clausus* policies are in fact less oriented toward mastery of course content. These deleterious effects are problematic for students in all disciplines; moreover, one might wonder about their specific consequences for fields leading to position of high social responsibility, such as for instance Medical Sciences, where professionals are expected to be highly motivated toward learning and to maintain up-to-date scientifically, so as to put this knowledge into practice. 

On the other hand, the ideological argument stating that *numerus clausus* favors a meritocratic system that selects the best students on the basis of their competence—and their competence only-[2], is challenged by two results. First, our findings call into question the *efficiency* of such a system. Despite its function to select the very best students, *numerus clausus* could paradoxically lead to a reduced level of learning. Indeed, in Study 3, we showed that when success at a task is subject to *numerus clausus* (vs. not having *numerus clausus*) short-term learning is reduced. Second, our findings cast doubt on the *fairness* of *numerus clausus*-based meritocratic systems. Supposedly, *numerus clausus* aims at establishing impartial and equitable “merit criteria” for selection, rather than “other criteria, such as wealth, sex, age, ethnic, or social status” ([18] p 8). Contrary to this, in Study 3, the deleterious effect of *numerus clausus* on learning was found to be especially prominent for disadvantaged groups. In our case, this disadvantaged group was women, to the extent that they were carrying out a stereotypically masculine task (i.e., a logical-mathematical task [71]) in competition with men, in a male-dominated field (i.e., STEM, [76,81]). Thus, the results of Study 3 might make one wonder to what extent the *numerus clausus* policy could contribute to the reproduction of social inequalities, thereby contradicting the equality principle in access to knowledge typical of Western democracies.

However, due to the practical reasons enounced in the introduction (e.g., demographic, economic), higher education institutions are regularly under pressure to resorting to *numerus clausus*. If *in-curriculum numerus clausus* has the paradoxical effects exposed in the present research, how then should higher education institutions cope with pressures to control the flow of their undergraduates? A look at the relevant literature shows that two main alternatives have generated some research: i) student selection *before* the entrance at university [78,79,82], and ii) student orientation [83]. As far as the first direction is concerned, applying *numerus clausus* before (i.e., *pre-curriculum*) *versus* after (i.e, *in-curriculum*) entering higher education has been described as enabling students to save time (by enhancing subsequent students’ success rate and lowering the drop-out rate, [84]), and governments to save money (by cutting the expenditure dedicated to cover the excessive numbers of students [85]). Despite these practical advantages, the results of the present research suggest that *pre-curriculum numerus clausus* may anticipate the pernicious effects of *in-curriculum numerus clausus*, weakening self-efficacy beliefs, mastery goals, and learning process of high-school students or other candidates aiming at passing higher education entrance examinations. As far as the second direction is concerned, other authors have stressed the importance of developing secondary–tertiary education partnership so as to improve school counseling practices and students’ orientation programs [83]. Vocational guidance helps pre-college students to refine and/or adapt their academic and career ambitions by clarifying their goals and preferences [86,87], thereby reducing the likelihood of choosing a curriculum for extrinsic reasons (because it is popular, easy, conducive to desirable positions, etc.). By rationalizing high-school students’ college choices, an optimal school counseling could regulate the flow of higher education applicants and, eventually, reduce the need for a stern *numerus clausus* policy. Although more research is needed to address this issue, it seems reasonable to assume that a counseling system informed by research on the promotion of self-efficacy and mastery goals could offer a viable alternative to *numerus clausus*.

Some limitations should be mentioned. The two first studies only revealed evidence of a link between *numerus clausus*, self-efficacy and mastery goals with Swiss students. Although there is no reason to expect different results in other Western industrialized societies (competition is—at least for individualistic countries—similarly perceived by the majority of their citizens [31]), replications in other countries would be an important endeavor for the future. More importantly, long-term effects of in-curriculum *numerus clausus* should be investigated so as to determine to what extent first-year selection policies influence individuals’ motivation and learning throughout their entire university curriculum and—eventually—in their professional practice. Furthermore, our data do not allow us to indicate whether the effects of *numerus clausus* are similar among low and high achievers or not. Therefore, both a longitudinal design and a model including the students’ initial ability could represent appealing directions for future investigations. Finally, as far as Study 3 is concerned, poor external validity, as well as the small effect size of the total effect, prevent us from claiming that the effect of *numerus clausus* on learning is replicable in a more ecological context. Thus, additional field investigations are needed in this area.

Despite these limitations, the present article contributes to a better understanding of the role and effects of *numerus clausus*, one of the most widely debated selection processes in higher education. Selection in higher education has always existed, and it is interesting to note that the very first university in Europe, established in Athens by the Roman emperor Marcus Aurelius in 161 A.D., aimed at selecting the future high-ranking official of the governmental apparatus [88]. Nowadays, as we have seen, *numerus clausus* works as a tool for selection of the political, economic and intellectual élites [89]. However, our research shows that such *numerus clausus* policy is not without consequences on self-efficacy beliefs, mastery goals and competence acquisition of students, i.e., tomorrow’s medical doctors, schoolteachers, or administrative officers. In sum, our results underline the importance that the selective function of higher education does not conflict with its formative function [90], as well as the importance that the selection process does not impair the learning process, and that the endeavor of selecting the most competent students does not—paradoxically—lead students to invest less in learning.
